# Neutrophils in Oral Paracoccidioidomycosis and the Involvement of Nrf2

**DOI:** 10.1371/journal.pone.0076976

**Published:** 2013-10-24

**Authors:** Vera Cavalcanti Araújo, Ana Paula Dias Demasi, Andresa Borges Soares, Fabrício Passador-Santos, Marcelo Henrique Napimoga, Elizabeth Ferreira Martinez, Nadir Severina Freitas, Ney Soares Araújo

**Affiliations:** Department of Pathology, São Leopoldo Mandic Institute and Research Center, Campinas, São Paulo, Brazil; Warren Alpert Medical School of Brown University, United States of America

## Abstract

Neutrophils have been implicated in granuloma formation in several infectious diseases, in addition to their main phagocytic and pathogen destruction role. It has been demonstrated that Nrf2 regulates antioxidant protection in neutrophils, attenuating inflammation without compromising the hosts bacterial defense. In this study, we analyzed the presence of neutrophils in *Paracoccidioides brasiliensis* mycosis (PCM), as well as the immunoexpression of Nrf2. Thirty-nine cases of oral PCM were classified according to quantity of fungi and to the presence of loose or well-organized granulomas and microabscesses. An Nrf2 antibody was used for immunohistochemical analysis. The results showed that neutrophils are present in microabscesses and loose granulomas, but were absent in structured granulomas. A greater quantity of fungi was shown in cases with only loose granulomas when compared to loose and well organized granulomas. Nrf2 was observed in the nuclei of neutrophils of loose granulomas and abscesses, with its expression in loose granulomas maintained despite the additional presence of well organized granulomas in the same specimen. This study suggests that neutrophils participate in *P. brasiliensis* granuloma formation and that Nrf2 has a possible role in neutrophil survival, via modulation of the inflammatory response.

## Introduction

Paracoccidioidomycosis (PCM) is known to be one of the most frequent systemic fungal infections affecting the rural population of Latin America, predominantly in Brazil. PCM is caused by the thermally dimorphic fungus, *Paracoccidioides brasiliensis* (*P. brasiliensis*) [1]. The disease is characterized by a chronic inflammatory granulomatous reaction, which is the consequence of a Th-1 mediated adaptive immune response. The immune response begins with a contact between *P. brasiliensis* and the host tissue, leading to a subsequent accumulation of neutrophils and microabscess formation. As the lesion progresses, the neutrophils are replaced by macrophages and multinucleated giant cells, followed by epithelioid cells. These cells are concomitantly found within the formation of a mononuclear cell halo. Fibrosis, of varying intensity, is generally seen surrounding the granuloma, which is gradually replaced by fibrous scar tissue [2]. This morphology is normally seen in a well-organized granuloma, however, depending on the immunological response of the host, it may also be observed in ill-defined granuloma, or so-called loose granuloma (Figure S1).

Oral lesions are common in PCM, occurring in more than 50 % of cases, and may affect the gingiva, buccal mucosa, hard palate, lips and tongue [3]. They arise as ulcerated lesions with a dotted, vascular pattern over a granulomatous base. Cytological analyses of mucosal lesions have been performed. The presence of macrophages and CD4 lymphocytes has been shown in compact granulomas, whilst eosinophils, CD20 lymphocytes, plasma and mast cells in non-granulomatous areas [4]. Neutrophils are found in microabscesses. More recently, regulatory T cells have also been shown in oral lesions [5].

In infectious diseases, the function of neutrophils, as efficient phagocytic cells, has been demonstrated in a variety of experimental models, including *Listeria* [6], *Legionella pneumophila* [7], *Mycobacterium* [8], *Candida albicans* [9] *Toxoplasma gondii* [10] and *Trypanosoma cruzi* [11]. Neutrophils are amongst the first cells to be recruited to the site of any inflammatory insult, where their activation initiates the oxidative burst, producing reactive oxygen species (ROS). In addition, protease enzymes and other pro-inflammatory mediators are released by degranulation following activation. While essential for microbial killing, ROS and proteases damage cells and extracellular surrounding biological substrates, whereas the cytokine release stimulates the influx of other inflammatory cells [12]. Current evidence has shown that neutrophils are involved in mechanisms other than phagocytosis, including initiation of granuloma organization [6]. Neutrophils express an assortment of cytokines, which are crucial to their role in innate and adaptive immune responses, however, if imbalanced, they may also be determinants of chronic inflammation [13]. Therefore, modulation of neutrophil activity/lifespan is crucial so that pathogens are destroyed, tissue injury is constrained and inflammation can be resolved.

Recently, Kong et al. [14] demonstrated that nuclear factor erythroid-2-related factor-2 (Nrf2), a basic Leucine Zipper (bZIP) transcription factor, operates as a critical immunomodulator in leucocytes, including neutrophils that improve host survival during sepsis [14]. Nfr2 has been regarded as a master regulator of the cellular redox state. It coordinates the induction of antioxidant enzymes and associated defenses, such as glutathione biosynthesis and regeneration of reduced NADP [15]. Nrf2 is found in the cytoplasm and is associated with the actin-anchored protein Keap1. Under oxidative and electrophilic stress conditions, Nrf2 is released from Keap1 and translocates to the nucleus, where it binds to a *cis*-element referred to as antioxidant response element (ARE). It then activates the expression of several cytoprotective genes and inhibits oxidative tissue injury, therefore, protecting against persistent inflammation [15].

Motivated by the aforementioned findings, the aims of this study were to assess for the presence of neutrophils in *P. brasiliensis* infection in oral tissues, and analyze immunoexpression of Nrf2 in these cells.

## Materials and Methods

### Materials

Thirty-nine cases of PCM from oral biopsies between 2004 and 2012 were retrieved from the archives of the Oral Pathology Laboratory, São Leopoldo Mandic Institute and Research Center. The Ethics Committee of São Leopoldo Mandic Institute and Research Center approved the study. All biopsies were accompanied by signed informed consent from the patient. The biopsies were fixed in neutral formalin and embedded in paraffin. Hematoxylin (Dinâmica, Diadema, Brazil) and Eosin (H&EMerck, Darmstadt, Germany), and Periodic Acid-Schiff (PAS, Merck, Darmstadt, Germany) stained slides were reviewed and classified according to the quantity of fungi and presence of loose or well-organized granulomas and microabscesses. Well-organized granulomas were characterized by central macrophages, multinucleated giant cells and epithelioid cells, surrounding by lymphocytes, fibroblasts, and finally fibrosis in very structured nodules. Loose granulomas were characterized by diffuse foci of macrophages, neutrophils, epithelioid and plasma cells, eosinophils, multinucleated giant cells without structural organization. The entire section was screened, and the area of greatest fungal load (hotspot) was selected for quantification, using high power microscopy (400X). After reviewing all of the cases, 10 fungi were set as the division marker for low or high PB quantity. If fewer than 10 were found, low fungi quantity was attributed to the case. If 10 or more were found, a high quantity was attributed.

### Immunohistochemistry

One paraffin block from each case was selected for the immunohistochemical study of Nrf2.

Five µm sections were deparaffinized, hydrated and immersed in 3% hydrogen peroxide for 30 minutes (Dinâmica, Diadema, SP, Brazil). Antigen retrieval was achieved by boiling the slides in a steamer immersed in a citrate buffer (pH 6.0, 1 hour (Sigma, St Louis, MO, USA). Subsequently, the sections were incubated at 4°C with the primary antibody (Nrf2, 1∶100 from Santa Cruz Biotechnology, catalog number # Sc7313) overnight and then with the biotinylated secondary antibody peroxidase conjugated streptavidin system (LSAB, Dako, Carpinteria, CA, USA) for 1h at 37°C. The sections were stained for 5 min at 37°C with 3.3′- diaminobenzidine tetrahydrochloride (DAB, Dako, Carpinteria, CA, USA) and counter-stained with hematoxylin (Dinâmica, Diadema, Brazil). Human lung carcinoma was used as positive control and negative control was achieved by omitting the primary antibody.

### Staining Evaluation

Using a double-headed microscope (Olympus BX51, Tokyo, Japan), two examiners interpreted the immunohistochemical reactions for Nrf2. The labeled sections were evaluated qualitatively and semi-quantitatively. Nrf2 was evaluated in abscesses and granulomas where neutrophils were present. Nuclear expression alone was considered positive. Expression scores were assigned according to the percentage of nuclear positive cells, from 0 to 2 (0, negative; 1, less than 50%; 2, 50% or more than 50% of nuclear positive cells).

Digital photomicrographs were obtained on a Zeiss Axioskop 2 plus microscope equipped with an Axiocam digital camera and AxioVision application software (Carl Zeiss, Gottingen, Germany).

### Statistical analysis

Statistical analysis was performed using the Chi-squared and Mann-Whitney tests for quantity of fungi and Nrf2 immunoexpression, respectively, in cases with loose granulomas exclusively, compared to those with loose plus well-organized granulomas. For both tests, GraphPad Prism 4.0 (La Jolla, CA, USA) software was used, with a significance level of 5%.

### Results

**Figure 1:**
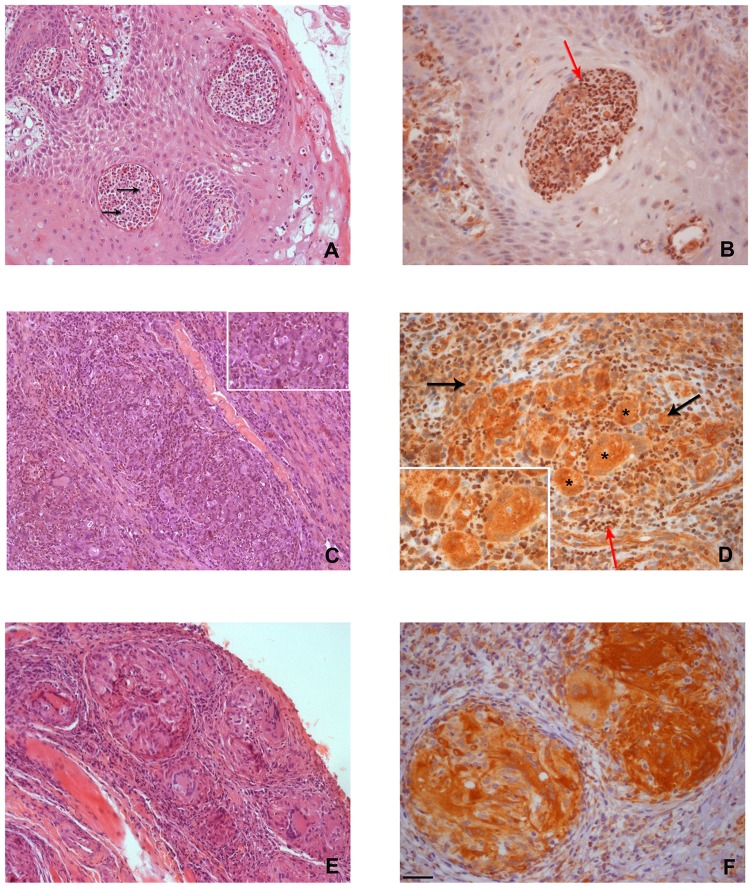
Histological and *Nrf2* immunoexpression in oral *Paracoccidioides brasiliensis* mycosis. (A) Presence of a collection of neutrophils and rare macrophages forming microabscesses (black arrows) – H&E. (B) Microabscesses showing Nrf2 nuclei expression in neutrophils (red arrow). (C) Loose granuloma displaying a mixture of neutrophils, macrophages, multinucleated giant cells and epithelioid cells. Inset: numerous *P. brasiliensis* are shown – H&E. (D) Nuclei Nrf2 expression highlighting the presence of neutrophils (red arrow), cytoplasm of multinucleated giant cells (asterisk) and cytoplasm of macrophages (black arrows) in loose granulomas. (E) Structured granuloma constituted by macrophages, multinucleated giant cells and epithelial cells surrounded by fibrosis- H&E (F) Cytoplasmic Nrf2 expression in multinucleated giant cells and macrophages in well-organized granulomas. Note the absence of neutrophils in these structures. Bars: (A), (C) and (E)  = 45 µm; (B), (D) and (F)  = 25 µm.

Histological review of the mucosal lesions showed evidence of pseudo-epitheliomatous hyperplasia and numerous microabscesses. In addition to well-organized and loose granulomas, the underlying lamina propria presented a diffuse inflammatory infiltrate with lymphocytes, plasma cells, macrophages and multinucleated giant cells (Figures 1A, 1C, 1E).

**Figure 2:**
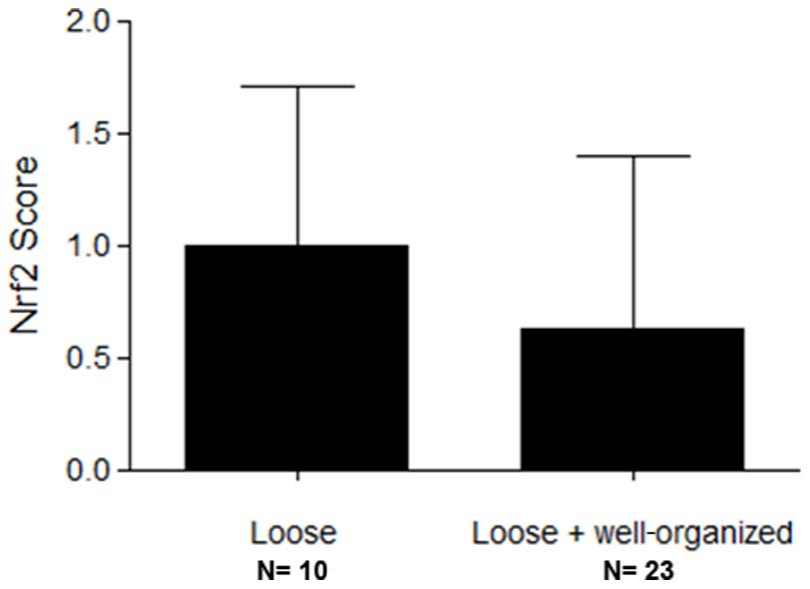
Immunohistochemical expression of Nrf2. The score of Nrf2 was higher in the loose granuloma when compared to loose plus well-organized granuloma, although there was no significant difference (p = 0.17; Mann-Whitney). Data are represented as mean ±SD.

The characteristics of the studied samples are described in Table 1. For each case, the quantity of microorganisms, the presence of loose or well-organized granulomas and microabscesses, and Nrf2 positivity in neutrophils are also shown. Twenty-three of the 39 cases analyzed (58.9%) presented both well-organized and loose granulomas, ten (25.6%) presented loose granulomas only and three (7.6%) presented well-organized granulomas only. In three cases, no granulomas were detected. Microabscesses were observed in 25 cases (64.1%). We observed that neutrophils were present in both microabscesses and loose granulomas, and absent in well-organized granulomas (Figures 1A, 1C, 1E). The statistical analysis revealed a greater quantity of fungi in cases with loose granulomas only when compared with those with loose plus well-organized granulomas (p = 0,0005, Chi-square test) (Table 2, Figure 2).

**Table 1:** Quantity of fungus, presence of microabscesses, granuloma structure and positivity to Nrf2 in cases of oral *Paracoccidioides brasiliensis* mycosis.

		*Well-organized granuloma*		*Loose granuloma*		*Microabscesses*	
Case	quantity of fungi	HE	NRF2	HE	NRF2	HE	NRF2
1	high	present	0	absent		present	0
2	high	absent		absent		present	0
3	high	present	0	present	0	present	0
4	high	absent		absent		present	1
5	high	absent		present	0	absent	
6	high	absent		present	2	absent	
7	high	absent		present	2	present	2
8	high	absent		present	1	present	2
9	high	absent		present	1	present	1
10	high	absent		present	1	present	1
11	high	absent		absent		present	0
12	high	absent		present	0	present	0
13	small	present	0	present	0	absent	
14	small	present	0	present	0	present	1
15	small	present	0	present	1	present	1
16	small	present	0	present	1	present	2
17	small	present	0	present	2	present	2
18	small	present	0	present	1	present	2
19	small	absent		present	1	present	1
20	small	present	0	present	2	present	2
21	small	present	0	present	2	absent	0
22	small	present	0	present	1	present	2
23	small	absent	0	present	0	absent	0
24	small	present	0	present	1	absent	0
25	small	present	0	present	0	present	1
26	small	present	0	present	0	absent	
27	small	present	0	present	0	absent	
28	small	absent		present	0	present	0
29	small	present	0	present	0	present	0
30	small	present	0	present	0	present	1
31	small	present	0	present	0	present	0
32	small	present	0	present	0	present	2
33	small	present	0	absent		absent	
34	small	present	0	present	0	absent	
35	small	present	0	absent		absent	
36	small	present	0	present	0	absent	
37	small	present	0	present	0	absent	
38	small	present	0	present	0	present	0
39	small	present	0	present	0	absent	

**Table 2:** Quantity of fungi in biopsies containing paracoccidioidomycosis, exhibiting loose and loose plus well-organized granulomas.

	L	L+W	T
**High**	7	1	8
**Low**	3	22	25
**T**	10	23	33

L: Loose granuloma; L+W: Loose plus well-organized; T: Total.

The immunohistochemical analysis showed positivity to Nrf2 in the nuclei of neutrophils (Figures 1B, 1D, 1F). The intensity was homogenous and strong in all cells containing positive nuclei. In 16 cases (48.4%), Nrf2 was present in the nuclei of the neutrophils of loose granulomas, thus highlighting them amongst the cells that participated in granuloma formation (Figure 1D). The statistical analysis revealed that the immunohistochemical positivity to Nrf2 in the nuclei of neutrophils in loose granulomas was maintained even in the presence of well-organized granulomas within the same specimen (p = 0,17, Mann-Whitney test). In addition to the neutrophils, Nrf2 positivity was also observed in the cytoplasm of macrophages and multinucleated giant cells.

## Discussion

We have focused on the presence of neutrophils in the inflammatory response induced by the presence of the fungus *P. brasiliensis.* We analyzed human oral biopsies of patients with PCM. Neutrophils and macrophages were observed in microabscesses when *P. brasiliensis* was present. They were also seen in loose granulomas amongst other cells, including multinucleated giant cells and epithelioid cells.

Neutrophils are often the first cells to arrive at the scene of inflammation, migrating from the blood vessels to the affected tissue [12]. They are amongst the cells capable of recognizing and phagocytizing pathogens. Binding to *P. brasiliensis* occurs through neutrophil PRRs receptors, which recognize the pathogen-associated molecular patterns (PAMPs). These are molecules, which are expressed exclusively by microbes, thus are not found in higher organisms. They are efficient phagocytes that possess oxidative and non-oxidative mechanisms for microbial destruction. The former uses toxic reactive radicals (oxygen and nitrogen) in phagolysosoma, whilst the latter includes enzymes and antimicrobial peptides, such as defensins [12].

Despite the core importance of macrophages and T lymphocytes in the morphogenesis of the inflammatory process and the synergistic action of these cells in granuloma formation being accepted, [16] an immunoregulatory role for neutrophils has recently been demonstrated with several infectious diseases [9], [17]–[23]. This creates a microenvironment in which parasite specific T cells can be present. Therefore, the cytokines and chemokines produced by neutrophils can influence the development of the immune response to various microorganisms.

As expected, the biopsies with well-organized granulomas presented a lower quantity of fungi than the biopsies with loose granulomas. Furthermore, our statistical analysis has revealed that the quantity of fungi decreased from the loose, to the loose plus well-organized granulomas, indicating the course of the disease or possibly the immune status of the host. The number of neutrophils also decreased from loose to loose plus well-organized granulomas, and no neutrophils were found in those cases with well-organized granulomas exclusively. This scenario does not prove the participation of neutrophils in granuloma formation, however it is indeed suggestive of their participation [16] or alternatively this scenario may represent an ineffectual immune response. Regarding a possible role in granuloma formation, it was previously demonstrated in mice infected with *M. tuberculosis* that neutrophils are required for early structuring of the granuloma, presumably through secretion of chemokines such as CXCL-9/MIG by neutrophils. It was shown that depletion of neutrophils, neutralization of the chemokine or the use of CXCR3-deficient mice led to drastically reduced granuloma formation [24]. In addition, *Leishmania donovani*-infected mice, selectively depleted of neutrophils, displayed a delay in the maturation of hepatic granulomas and a decrease in inducible nitric oxide synthase expression within granulomas [23].

Activated neutrophils produce higher amounts of ROS than other inflammatory cells, which are toxic regardless of the cell type, including to the neutrophils themselves. Therefore, protection of neutrophils against their own toxic metabolites critically affects their survival and the progression of the inflammatory response. Our immunohistochemical results have shown that, in many cases, nuclei of neutrophils were Nrf2 positive. In order to be activated, Nrf2 must be released from Keap1 and translocated to the nucleus where it can signal the expression of several cytoprotective genes and inhibits oxidative tissue injury [15]. In macrophages and multinucleated giant cells, this transcription factor was only seen in the cytoplasm, indicating that it is not activated in these cells. This idea is supported by Goven et al. (2010), who investigated the association of primary spontaneous pneumothorax and oxidative stress in lung macrophages, and demonstrated that the induction of the antioxidant proteins was a consequence of the nuclear localization of the hypoxia inducible factor 1a (HIF-1a), but not of Nrf2. In fact, the organism possesses several systems to counteract oxidative stress [25].

The presence of Nrf2 was seen in the nuclei of neutrophils in microabscesses, as well as in the nuclei of those in the loose granulomas. This may indicate Nrf2-dependent up-regulation of antioxidant defenses at the crucial stages when neutrophils are the main effector killer cells of *P. brasiliensis*, i.e, in microabscesses, in the structuring of granulomas, or in cooperation with macrophages for the clearance of the parasite. Redox balance systems described in neutrophils include catalase, copper zinc superoxide dismutase and γ-glutamylcysteine synthase, all under Nrf2 transcriptional regulation via ARE sites [26].

In addition, ROS have been implicated as common signal transducers for diverse stimuli that result in nuclear factor-kappaB (NF-κB) activation in inflammatory cells [27] and the maintenance of this activated state may drive the establishment of chronic inflammatory diseases [28]–[30]. Therefore, Nrf2-mediated induction of antioxidants in neutrophils may contribute to the redox modulation of the inflammatory response. In fact, disruption of Nrf2 in mice led to augmented lung inflammation during experimental sepsis [31]. Global gene expression profiling demonstrated that the main components of the innate immune response, including cytokines, CXC and CC chemokines, and cell adhesion molecules, were expressed at significantly higher levels in Nrf2 deficient lung cells when compared with wild type counterpart cells after LPS stimulation. In addition, many antioxidant genes were down regulated, and it was suggested that the pro-oxidant state of the cells could enhance LPS-induced NF-κB activation [31]. Moreover, impairment of cellular antioxidant defenses induction in mice by deletion of Nrf2 has been associated with enhanced susceptibility and severity of several inflammatory disorders, such as asthma, fibrosis, emphysema and colitis [32]–[34]. On the other hand, keap1 deletion, and the consequent enhancement of Nrf2 activity in neutrophils, markedly reduced mortality, organ injury, circulating levels of inflammatory mediators and bacteremia in a mouse experimental model of sepsis, without compromising host bacterial defense mechanism [14].

Through the histological investigation of the inflammatory reaction against oral *P. brasiliensis* infection, this study has shown that neutrophils were present in microabscesses and in loose granulomas, however, they were absent as the granulomas became more structured, suggesting that neutrophils participate in *P. brasiliensis* granuloma formation despite the control of fungal load. We have also demonstrated Nrf2 positivity in a greater number of neutrophils, which may indicate an attempt by these cells to reinforce their survival by increasing antioxidant resistance, which might diminish tissue injury, as well as modulate the inflammatory response.

## Supporting Information

Figure S1 **Paracoccidioidomycosis.** A loose granuloma showing many fungi and nuclear Nrf2 immunostaining highlighting the neutrophils. Original magnification 1000X.
(TIF)

## References

[1] Franco M, Peracoli MT, Soares A, Montenegro R, Mendes RP, et al (1993) Host-parasite relationship in paracoccidioidomycosis. Curr Top Med Mycol 5: 115–149. 8242798

[2] Fortes MR, Miot HA, Kurokawa CS, Marques ME, Marques SA (2011) Immunology of paracoccidioidomycosis. An Bras Dermatol 86: 516–524. 21738969 10.1590/s0365-05962011000300014

[3] Ameen M, Talhari C, Talhari S (2010) Advances in paracoccidioidomycosis. Clin Exp Dermatol 35: 576–80. 19874328 10.1111/j.1365-2230.2009.03647.x

[4] Kaminagakura E, Bonan PR, Jorge J, Almeida OP, Scully C (2007) Characterization of inflammatory cells in oral paracoccidioidomycosis. Oral Dis 13: 434–439. 17577332 10.1111/j.1601-0825.2006.01319.x

[5] Pagliari C, Fernandes ER, Stegun FW, da Silva WL, Seixas Duarte MI, et al (2011) Paracoccidioidomycosis: cells expressing IL17 and Foxp3 in cutaneous and mucosal lesions. Microb Pathog 50: 263–267. 21296652 10.1016/j.micpath.2010.12.008

[6] Appelberg R, Castro AG, Silva MT (1994) Neutrophils as effector cells of T-cell-mediated, acquired immunity in murine listeriosis. Immunology 83: 302–307. 7835951 PMC1414945

[7] Tateda K, Moore TA, Deng JC, Newstead MW, Zeng X, et al (2001) Early recruitment of neutrophils determines subsequent T1/T2 host responses in a murine model of Legionella pneumophila pneumonia. J Immunol 166: 3355–3361. 11207291 10.4049/jimmunol.166.5.3355

[8] Pedrosa J, Saunders BM, Appelberg R, Orme IM, Silva MT, et al (2000) Neutrophils play a protective nonphagocytic role in systemic Mycobacterium tuberculosis infection of mice. Infect Immun 68: 577–583. 10639420 10.1128/iai.68.2.577-583.2000 PMC97179

[9] Romani L, Mencacci A, Cenci E, Del Sero G, Bistoni F, et al (1997) An immunoregulatory role for neutrophils in CD4+ T helper subset selection in mice with candidiasis. J Immunol 158: 2356–2362. 9036985

[10] Marshall AJ, Denkers EY (1998) Toxoplasma gondii triggers granulocyte-dependent cytokine-mediated lethal shock in D-galactosamine-sensitized mice. Infect Immun 66: 1325–1333. 9529049 10.1128/iai.66.4.1325-1333.1998 PMC108056

[11] Chen L, Watanabe T, Watanabe H, Sendo F (2001) Neutrophil depletion exacerbates experimental Chagas' disease in BALB/c, but protects C57BL/6 mice through modulating the Th1/Th2 dichotomy in different directions. Eur J Immunol 31: 265–275. 11265643 10.1002/1521-4141(200101)31:1<265::AID-IMMU265>3.0.CO;2-L

[12] Kennedy MA (2010) A brief review of the basics of immunology: the innate and adaptive response. Vet Clin North Am Small Anim Pract 40: 369–379. 20471522 10.1016/j.cvsm.2010.01.003

[13] Mantovani A, Cassatella MA, Costantini C, Jaillon S (2011) Neutrophils in the activation and regulation of innate and adaptive immunity. Nat Rev Immunol 11: 519–531. 21785456 10.1038/nri3024

[14] Kong X, Thimmulappa R, Craciun F, Harvey C, Singh A, et al (2011) Enhancing Nrf2 pathway by disruption of Keap1 in myeloid leukocytes protects against sepsis. Am J Respir Crit Care Med 184: 928–938. 21799073 10.1164/rccm.201102-0271OC PMC3208662

[15] Copple IM (2012) The Keap1-Nrf2 cell defense pathway – a promising therapeutic target? Adv Pharmacol 63: 43–79. 22776639 10.1016/B978-0-12-398339-8.00002-1

[16] Appelberg R (2007) Neutrophils and intracellular pathogens: beyond phagocytosis and killing. Trends Microbiol 15: 87–92. 17157505 10.1016/j.tim.2006.11.009

[17] Bliss SK, Marshall AJ, Zhang Y, Denkers EY (1999) Human polymorphonuclear leukocytes produce IL-12, TNF-alpha, and the chemokines macrophage-inflammatory protein-1 alpha and -1 beta in response to Toxoplasma gondii antigens. J Immunol 162: 7369–7375. 10358188

[18] Bennouna S, Bliss SK, Curiel TJ, Denkers EY (2003) Cross-talk in the innate immune system: neutrophils instruct recruitment and activation of dendritic cells during microbial infection. J Immunol 171: 6052–6058. 14634118 10.4049/jimmunol.171.11.6052

[19] Tsuda Y, Takahashi H, Kobayashi M, Hanafusa T, Herndon DN, et al (2004) Three different neutrophil subsets exhibited in mice with different susceptibilities to infection by methicillin-resistant *Staphylococcus aureus* . Immunity 21: 215–226. 15308102 10.1016/j.immuni.2004.07.006

[20] Ribeiro-Gomes FL, Otero AC, Gomes NA, Moniz-De-Souza MC, Cysne-Finkelstein L, et al (2004) Macrophage interactions with neutrophils regulate Leishmania major infection. J Immunol 172: 4454–4462. 15034061 10.4049/jimmunol.172.7.4454

[21] Allenbach C, Zufferey C, Perez C, Launois P, Mueller C, et al (2006) Macrophages induce neutrophil apoptosis through membrane TNF, a process amplified by Leishmania major. J Immunol 176: 6656–6664. 16709824 10.4049/jimmunol.176.11.6656

[22] Charmoy M, Megnekou R, Allenbach C, Zweifel C, Perez C, et al (2007) Leishmania major induces distinct neutrophil phenotypes in mice that are resistant or susceptible to infection. J Leukoc Biol 82: 288–299. 17449725 10.1189/jlb.0706440

[23] McFarlane E, Perez C, Charmoy M, Allenbach C, Carter KC, et al (2008) Neutrophils contribute to development of a protective immune response during onset of infection with Leishmania donovani. Infect Immun 76: 532–541. 18056477 10.1128/IAI.01388-07 PMC2223441

[24] Seiler P, Aichele P, Bandermann S, Hauser AE, Lu B, et al (2003) Early granuloma formation after aerosol Mycobacterium tuberculosis infection is regulated by neutrophils via CXCR3-signaling chemokines. Eur J Immunol 33: 2676–2686. 14515251 10.1002/eji.200323956

[25] Goven D, Boutten A, Leçon-Malas V, Marchal-Sommé J, Soler P, et al (2010) Induction of heme oxygenase-1, biliverdin reductasa and H-ferritin in lung macrophages in smokers with primary spontaneous pneumothorax: role of HIF-1 alpha. PLoS One 28 5(5): e10886. 10.1371/journal.pone.0010886 PMC2878337 20526373

[26] Kinnula VL, Soini Y, Kvist-Mäkelä K, Savolainen ER, Koistinen P (2002) Antioxidant defense mechanisms in human neutrophils. Antioxid Redox Signal 4: 27–34. 11970840 10.1089/152308602753625825

[27] Kaul N, Forman HJ (1996) Activation of NF kappa B by the respiratory burst of macrophages. Free Radic Biol Med 21: 401–405. 8855453 10.1016/0891-5849(96)00178-5

[28] Tergaonkar V (2006) NFkappaB pathway: a good signaling paradigm and therapeutic target. Int J Biochem Cell Biol 38: 1647–1653. 16766221 10.1016/j.biocel.2006.03.023

[29] de Visser KE, Eichten A, Coussens LM (2006) Paradoxical roles of the immune system during cancer development. Nat Rev Cancer 6: 24–37. 16397525 10.1038/nrc1782

[30] Karin M (2006) Nuclear factor-kappaB in cancer development and progression. Nature 441: 431–436. 16724054 10.1038/nature04870

[31] Thimmulappa RK, Lee H, Rangasamy T, Reddy SP, Yamamoto M, et al (2006) Nrf2 is a critical regulator of the innate immune response and survival during experimental sepsis. J Clin Invest 116: 984–995. 16585964 10.1172/JCI25790 PMC1421348

[32] Rangasamy T, Cho CY, Thimmulappa RK, Zhen L, Srisuma SS, et al (2004) Genetic ablation of Nrf2 enhances susceptibility to cigarette smoke-induced emphysema in mice. J Clin Invest 114: 1248–1259. 15520857 10.1172/JCI21146 PMC524225

[33] Rangasamy T, Guo J, Mitzner WA, Roman J, Singh A, et al (2005) Disruption of Nrf2 enhances susceptibility to severe airway inflammation and asthma in mice. J Exp Med 202: 47–59. 15998787 10.1084/jem.20050538 PMC2212893

[34] Kensler TW, Wakabayashi N, Biswal S (2007) Cell survival responses to environmental stresses via the Keap1-Nrf2-ARE pathway. Annu Rev Pharmacol Toxicol 47: 89–116. 16968214 10.1146/annurev.pharmtox.46.120604.141046

