# Supplementary figures and images for: Neutrophils in Oral Paracoccidioidomycosis and the Involvement of Nrf2
Source: PLoS One. 2013 Oct 24;8(10):e76976. doi: 10.1371/journal.pone.0076976 (PMC3811996; doi:10.1371/journal.pone.0076976)

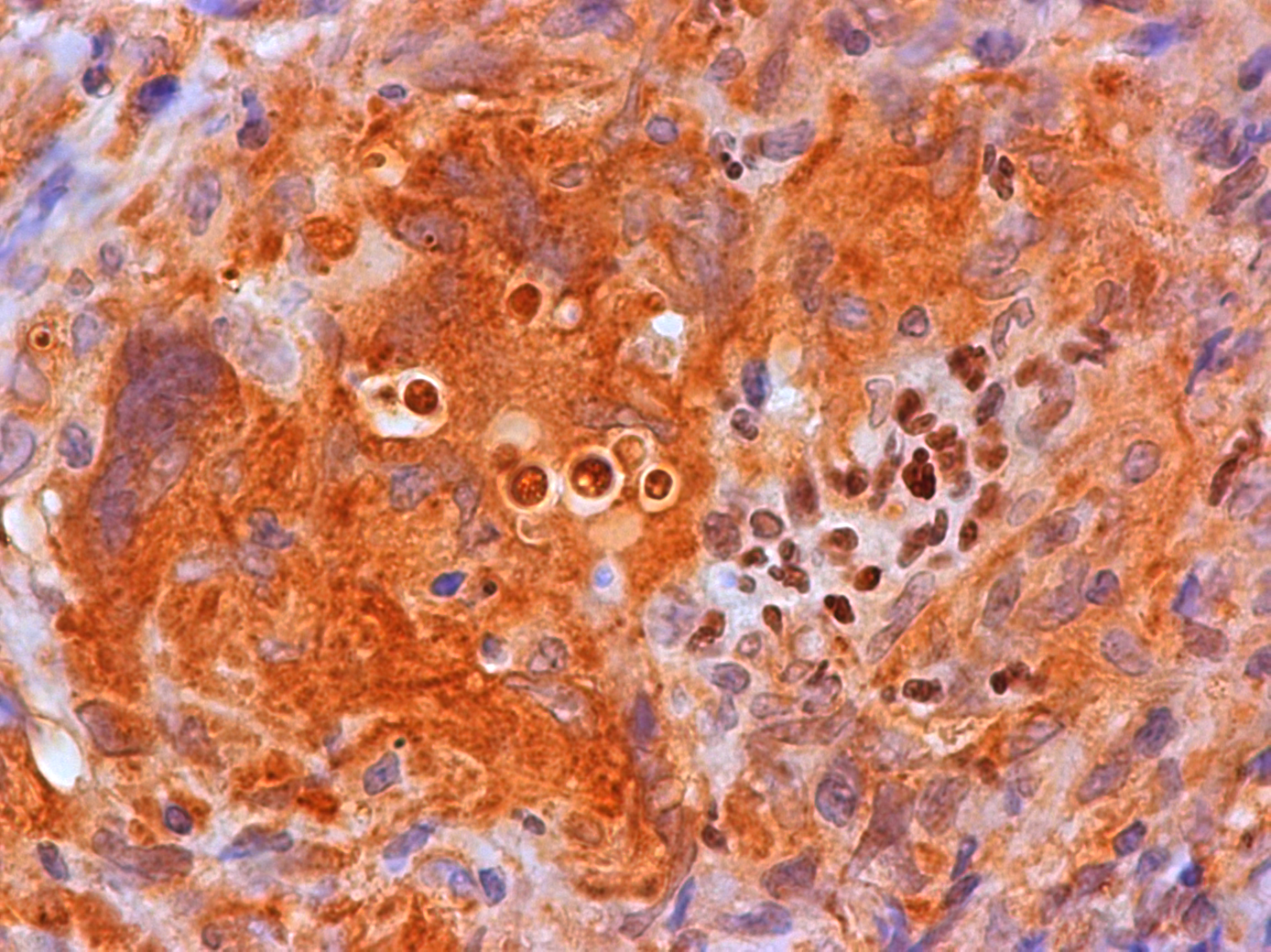

Supplement: Figure S1 — Paracoccidioidomycosis. A loose granuloma showing many fungi and nuclear Nrf2 immunostaining highlighting the neutrophils. Original magnification 1000X. (TIF) [file pone.0076976.s001.tif]
